# Synthesis, spectroscopic and Hirshfeld surface analysis and fluorescence studies of (2*E*,2′*E*)-3,3′-(1,4-phenyl­ene)bis­[1-(4-hy­droxy­phen­yl)prop-2-en-1-one] *N*,*N*-di­methyl­formamide disolvate

**DOI:** 10.1107/S2056989018007429

**Published:** 2018-05-22

**Authors:** Huey Chong Kwong, Ai Jia Sim, C. S. Chidan Kumar, Ching Kheng Quah, Suchada Chantrapromma, S. Naveen, Ismail Warad

**Affiliations:** a School of Chemical Sciences, Universiti Sains Malaysia, Penang 11800 USM, Malaysia; b X-ray Crystallography Unit, School of Physics, Universiti Sains Malaysia, 11800 USM, Penang, Malaysia; c Department of Engineering Chemistry, Vidya Vikas Institute of Engineering & Technology, Visvesvaraya Technological University, Alanahally, Mysuru 570028, Karnataka, India; d Department of Chemistry, Faculty of Science, Prince of Songkla University, Hat-Yai, Songkhla 90112, Thailand; e Department of Physics, School of Engineering and Technology, Jain University, Bangalore 562 112, India; f Department of Chemistry, Science College, An-Najah National University, PO Box 7, Nablus, West Bank, Palestinian Territories

**Keywords:** bis­chalcone, spectroscopy, centrosymmetric, Hirshfeld surface, fluorescence, crystal structure

## Abstract

In the bis­chalcone mol­ecule of the title compound, C_24_H_18_O_4_·2C_3_H_7_NO, the central benzene and terminal hy­droxy­phenyl rings form a dihedral angle of 14.28 (11)° and the central C=C double bond adopts a *trans* configuration. In the crystal, the bis­chalcone and solvate mol­ecules are inter­connected *via* O—H⋯O hydrogen bonds, which were investigated by Hirshfeld surface analysis. Solid-state fluorescence was measured at λ_ex_ = 4400 Å. The emission wavelength appeared at 5510 Å, which corresponds to yellow light and the solid-state fluorescence quantum yield (*F*_f_) is 0.18.

## Abstract 2

In the bis­chalcone mol­ecule, the central benzene and terminal hy­droxy­phenyl rings form a dihedral angle of 14.28 (11)° and the central C=C double bond adopts a *trans* conformation. In the crystal, the title mol­ecule and solvate are linked by O—H⋯O hydrogen bonds.

## Chemical context   

The development of new fluorescent probes has attracted much attention because of their applications in a wide range of electronic and optoelectronic devices related to telecommunications, optical computing, optical storage and optical information processing. Fluorescence generally occurs when a fluorescent probe (fluoro­phore) resonantly absorbs electromagnetic radiation that promotes it to an excited electronic state; subsequent relaxation of the excited state results in the emission of light, in which a portion of the excitation energy is lost through heat or vibration, and the rest is emitted at longer wavelengths compared to the excitation radiation. For a given fluoro­phore, the fluorescence intensity is directly proportional to the intensity of the radiation received. Fluoro­phores can be identified and qu­anti­fied on the basis of their excitation and emission properties. Different materials may exhibit different colours and intensities of fluorescence despite seeming identical when observed in daylight conditions. In recent years, chalcones have been used in the field of material science as non-linear optical devices (Raghavendra *et al.*, 2017 ▸; Chandra Shekhara Shetty *et al.*, 2017 ▸), photorefractive polymers (Sun *et al.*, 1999 ▸), optical limiting (Shettigar *et al.*, 2006*a* ▸; Chandra Shekhara Shetty *et al.*, 2016 ▸) and electrochemical sensing agents (Delavaux-Nicot *et al.*, 2007 ▸). The α,β-unsaturated ketone (C=C—C=O) moiety in the chalcone skeleton plays a vital role in its biological activities (Kumar *et al.*, 2013*a* ▸,*b* ▸). Apart from these biological activities, the photophysical properties of chalcone derivatives have also attracted considerable attention from both chemists and physicists. In view of the above and as a part of our ongoing work on such mol­ecules (Shettigar *et al.*, 2006*b* ▸; Tejkiran *et al.*, 2016 ▸; Pramodh *et al.*, 2018 ▸; Naveen *et al.*, 2017 ▸), we herein report the synthesis, structure determination, Hirshfeld surface analysis and fluorescence properties of (2*E*,2′*E*)-3,3′-(1,4-phenyl­ene)bis­[1-(4-hy­droxy­phen­yl)prop-2-en-1-one] *N*,*N*-di­methyl­formamide disolvate.
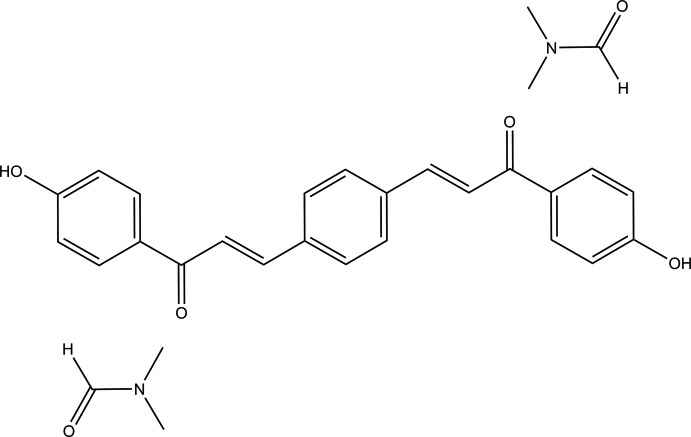


## Structural commentary   

**Figure 1:**
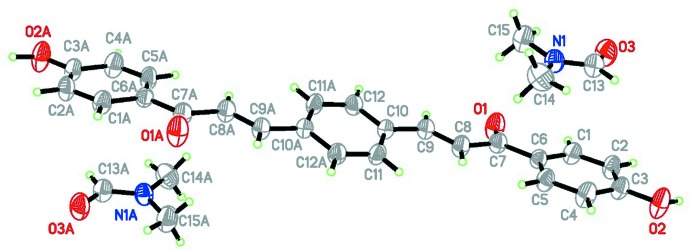
The mol­ecular structure of the title compound, showing the atom-labelling scheme, with 40% probability displacement ellipsoids. Atoms labelled with the suffix A are generated by the symmetry operation 1 − *x*, 2 − *y*, −*z*.

The asymmetric unit of the title compound comprises of half of the bis­chalcone mol­ecule, completed by inversion (symmetry operation 1 − *x*, 2 − *y*, −*z*) and a DMF mol­ecule (Fig. 1 ▸). The title compound crystallizes in the triclinic system with *Z* = 1 in space group *P* 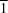. The bis­chalcone mol­ecule is constructed from two individually planar rings (central benzene and terminal hy­droxy­phenyl rings) and a C=C—C(=O)—C enone bridge with the central C=C double bond in a *trans* configuration. The hy­droxy­phenyl (C1–C6) and benzene (C10–C12/C10*A*–C12*A*) rings are almost parallel to each other, subtending a dihedral angle of 14.28 (11)°. The enone fragment and its attached benzene ring are slightly twisted, as indicated by the torsion angles O1—C7—C8—C9 = −5.6 (4)° and C1—C6—C7—O1 = 1.7 (4)°. All bond lengths and angles of the titled compound are in normal ranges (Allen *et al.*, 2002 ▸).

## Supra­molecular features   

**Figure 2:**
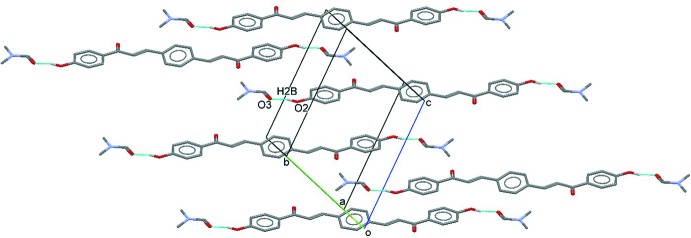
Partial crystal packing, showing the O—H⋯O hydrogen bonds (Table 1 ▸) between the bis­chalcone and DMF solvate mol­ecules.

In the crystal, the components are linked by O2—H2*B*⋯O3^i^ hydrogen bonds, which connect the DMF solvate mol­ecules to both terminal 4-hy­droxy­phenyl rings of the main mol­ecules (Fig. 2 ▸, Table 1 ▸).

## Database survey   

A search of the Cambridge Structural Database (CSD, Version 5.39, last update November 2016; Groom *et al.*, 2016 ▸) using (2*E*,2′*E*)-3,3′-(1,4-phenyl­ene)bis­(1-phenyl­prop-2-en-1-one) as main skeleton revealed the presence of four structures containing a similar bis-chalcone moiety to the title compound but with different substituents on the terminal phenyl rings, *viz*. 3,3′-(1,4-phenyl­ene)bis­[1-(*X*)prop-2-en-1-one], where *X* = 2-hy­droxy­phenyl (Gaur & Mishra, 2013 ▸), 4-chloro­phenyl (KIKFUG; Harrison *et al.*, 2007 ▸), 4-meth­oxy­phenyl (Harrison *et al.*, 2007*a* ▸) and 3,4-meth­oxy­phenyl (Harrison *et al.*, 2007*b* ▸). In these four compounds, the dihedral angles between the central and terminal phenyl ring are in the range 10.91–46.27°. In the positional isomer of the title compound, the 2-hy­droxy­phenyl moiety forms a dihedral angle of 10.91° with the benzene ring, compared to 14.28 (11)° in the title compound. The difference may arise from the intra­molecular hydrogen bond between 2-hy­droxy­phenyl unit and the adjacent carbonyl moiety.

## Hirshfeld surface analysis   

**Figure 3:**
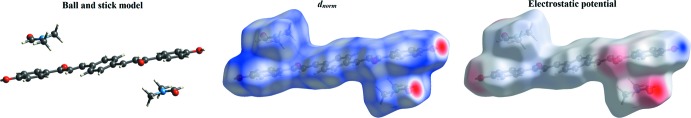
*d*_norm_ and electrostatic potential mapped on Hirshfeld surfaces to visualize the inter­molecular contacts in the title compound. The mol­ecule in the ball-and-stick model is in the same orientation as for the Hirshfeld surface and electrostatic potential plots.

Hirshfeld surface analysis (McKinnon *et al.*, 2004 ▸, 2007 ▸; Spackman & Jayatilaka, 2009 ▸; Spackman & McKinnon, 2002 ▸) was undertaken to qu­antify and give visual confirmation of the inter­molecular inter­action, and to explain the observed crystal structure. The *d*_norm_ surface plots, electrostatic potential and 2D fingerprint plots were generated by *CrystalExplorer 3.1* (Wolff *et al.*, 2012 ▸). The red spots on the *d*_norm_ surface arise as a result of the short inter­atomic contact; the positive electrostatic potential (blue regions) over the surface indicate hydrogen-donor potential, whereas the hydrogen-bond acceptors are represented by negative electrostatic potential (red regions). The *d*_norm_ surface plots and electrostatic potential of the title compound are shown in Fig. 3 ▸.

**Figure 4:**
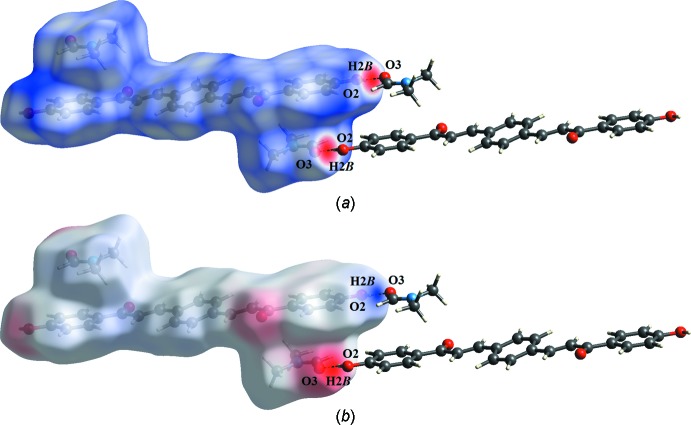
(*a*) *d*_norm_ and (*b*) electrostatic potential mapped on Hirshfeld surfaces in order to visualize the inter­molecular O—H⋯O inter­actions in the title compound.

**Figure 5:**
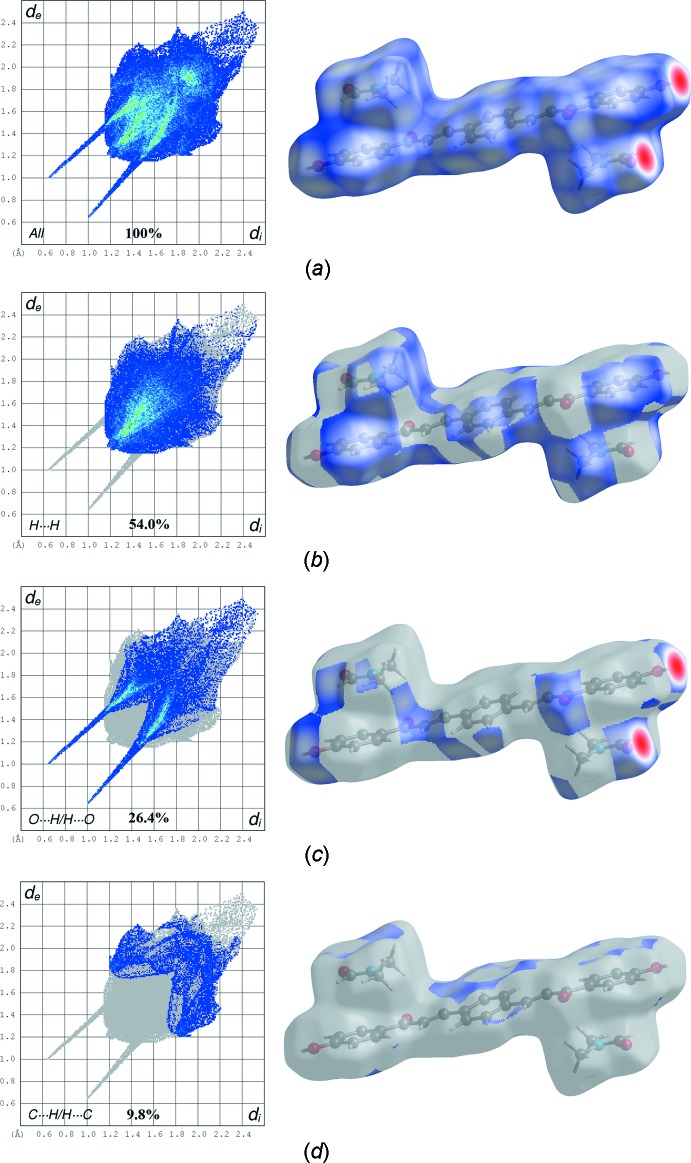
The two-dimensional fingerprint plots for the title compound showing contributions from different contacts; the views on the right highlight the relevant surface patches associated with the specific contacts.

**Figure 6:**
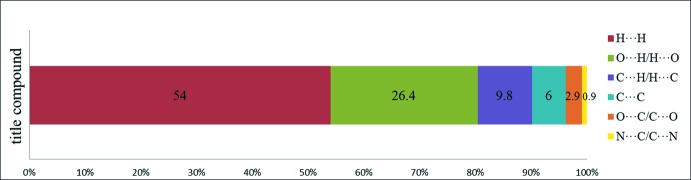
Percentage contributions of the various inter­molecular contacts contributing to the Hirshfeld surfaces of the title compound.

**Figure 7:**
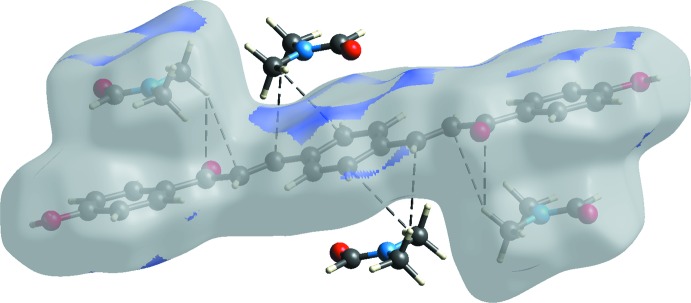
*d*_norm_ mapped on Hirshfeld surfaces to visualize the weak inter­molecular C—H⋯π and C—H⋯alkene inter­actions in the title compound.

The surface shows a red spot on the hydroxyl and carbonyl groups of the main mol­ecule and solvate, respectively. This is a result of the O2—H2*B*⋯O3 hydrogen bonds present in the structure (Fig. 4 ▸ *a*). These observations are further confirmed by the respective electrostatic potential map in which the atoms involved in the formation of hydrogen bonds are seen as blue (hydrogen-bond donor) and red (hydrogen-bond acceptor) spots (Fig. 4 ▸ *b*). The corresponding fingerprint plots (FP) for Hirshfeld surfaces show characteristic pseudo-symmetry wings in the *d_e_* and *d_i_* diagonal axes in the overall 2D FP (Fig. 5 ▸ *a*). H⋯H contacts (*i.e*. dispersive forces) make the greatest percentage contribution to the Hirshfeld surface, followed by O⋯H/H⋯O and C⋯H/H⋯C contacts (Fig. 6 ▸). The H⋯H contacts appear as the largest region on the fingerprint plot with a high concentration in the middle region, at *d*_e_ = *d*_i_ ∼ 1.2 Å with an overall contribution to the Hirshfeld surface of 54.0% (Fig. 5 ▸ *b*). The reciprocal O⋯H/H⋯O inter­action (26.4%) appears as two sharp symmetric spikes in the FP plot, which is characteristic of a strong hydrogen-bonding inter­action, at *d_e_* + *d_i_* ≃ 1.7 Å (Fig. 5 ▸ *c*). Two symmetrical broad blunted wings corresponding to the C⋯H/H⋯C inter­action (with a 9.8% contribution) appear at *d*_e_ + *d*_i_ ≃ 3.0 Å (Fig. 5 ▸ *d*). Analysis of the close contact on the *d*_norm_ surface plot suggests that the C⋯H/H⋯C inter­action might arise from weak C—H⋯π and C—H⋯alkene inter­actions between the solvate and main mol­ecules (Fig. 7 ▸).

## Solid-state fluorescence studies   

**Figure 8:**
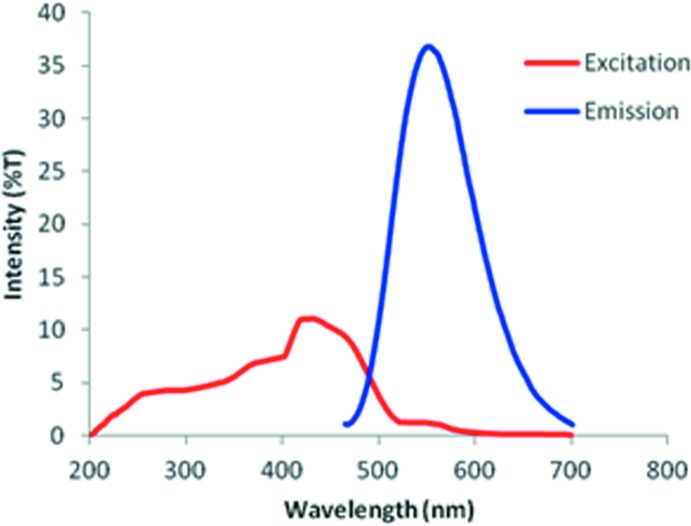
Solid-state excitation and emission spectrum for the title compound

A powder sample of the subject compound (0.72 mol) was heaped in the tray, covered with a quartz plate and was then fixed in the fluorescence spectrometer. The solid-state fluorescence properties were measured at the excitation wavelength (λ_ex_) of 4400 Å, which was selected from the absorption spectrum of the compound. The difference in the relative intensities of reflections between the sample and MgO powder was calibrated using diffusion reflections in a non-absorbed wavelength, in the present case this was 6500 Å. Finally, the fluorescence quantum yield (*F*_f_) was determined by Wrighton’s method and calculated according to the Φ_f_ = *j*_f_/(ϒ*j*_o_ − *j*) (Wrighton *et al.*, 1974 ▸) where, *j*_f_ is the fluorescence intensity of the sample, ϒ the calibration factor, *j*_0_ the back-scattered intensity of excitation light from a blank (here MgO) and *j* the back-scattered intensity of a loaded sample. The solid-state excitation and emission spectrum of the title compound (λ_ex_ at 4400 Å) is shown in Fig. 8 ▸. The emission wavelength (blue line) appears at 5510 Å, which corresponds to yellow light. The solid-state fluorescence quantum yield (*F*_f_) of the title compound is 0.18.

## Synthesis and crystallization   

A mixture of corresponding 4-hy­droxy­aceto­phenone 0.02 mol) and terephthaldi­aldehyde (0.01 mol) was dissolved in methanol (20 mL). A catalytic amount of NaOH was added to the solution dropwise with vigorous stirring. The reaction mixture was stirred for about 5–6 h at room temperature. The resultant crude product was filtered, washed successively with distilled water and recrystallized from acetone solution. Crystals suitable for X-ray diffraction studies were obtained by the slow evaporation technique using DMF as solvent. Yield: 85%, m.p. = 544–546 K.

FT–IR [ATR (solid) cm^−1^]: 3193 (O—H, ν), 3193 (Ar, C—H, ν), 2945 (methyl, C—H, νs), 2884 (methyl, C–H, ν), 1605 (C=O, ν), 1586, 1336 (Ar, C=C, ν), 1221 (C—O, ν), 1169 (C—N, ν). ^1^H NMR (500 MHz, DMSO): *δ* (ppm) 8.120–8.103 (*d*, 4H, *J* = 8.7 Hz, ^1^CH, ^5^CH), 8.028–7.997 (*d*, 2H, *J* = 15.6 Hz, ^8^CH), 7.964 (*s*, 4H, ^11^CH, ^12^CH), 7.737–7.706 (*d*, 2H, *J* = 15.6 Hz, ^9^CH), 6.931–6.914 (*d*, 4H, *J* = 8.7 Hz, ^2^CH, ^4^CH); ^13^C NMR (125 MHz, DMSO): *δ* ppm 187.05 (C7), 162.29 (C3), 141.86 (C9), 136.65 (C10), 131.28 (C1, C5), 129.92 (C6), 129.19 (C11, C12), 123.05 (C8), 115.39 (C2, C4).

## Refinement   

Crystal data, data collection and structure refinement details are summarized in Table 2 ▸. The O-bound H atom was located in a difference-Fourier map and refined freely. C–bound H atoms were positioned geometrically [C—H = 0.93–0.96 Å] and refined using a riding model with *U*_iso_(H) = 1.5*U*_eq_(C–meth­yl) and 1.2*U*_eq_(C) for other H atoms.

## Supplementary Material

Crystal structure: contains datablock(s) global, I. DOI: 10.1107/S2056989018007429/xu5924sup1.cif

Structure factors: contains datablock(s) I. DOI: 10.1107/S2056989018007429/xu5924Isup2.hkl

Supporting information file. DOI: 10.1107/S2056989018007429/xu5924Isup3.cml

CCDC reference: 1449629

Additional supporting information:  crystallographic information; 3D view; checkCIF report

## Figures and Tables

**Table 1:** Hydrogen-bond geometry (Å, °)

*D*—H⋯*A*	*D*—H	H⋯*A*	*D*⋯*A*	*D*—H⋯*A*
O2—H2*B*⋯O3^i^	0.99 (4)	1.63 (5)	2.592 (3)	162 (4)

Symmetry code: (i) 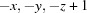.

**Table 2:** Experimental details

Crystal data	
Chemical formula	C_24_H_18_O_4_·2C_3_H_7_NO
*M*_r_	516.57
Crystal system, space group	Triclinic, *P* 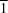
Temperature (K)	294
*a*, *b*, *c* (Å)	6.0569 (5), 9.5801 (5), 11.9941 (8)
α, β, γ (°)	72.867 (2), 84.649 (2), 86.710 (2)
*V* (Å^3^)	661.86 (8)
*Z*	1
Radiation type	Mo *K*α
μ (mm^−1^)	0.09
Crystal size (mm)	0.25 × 0.24 × 0.10

Data collection	
Diffractometer	Bruker APEXII DUO CCD area-detector
Absorption correction	Multi-scan (*SADABS*; Bruker, 2012 ▸)
*T*_min_, *T*_max_	0.961, 0.991
No. of measured, independent and observed [*I* > 2σ(*I*)] reflections	21963, 3039, 1944
*R*_int_	0.043
(sin θ/λ)_max_ (Å^−1^)	0.650

Refinement	
*R*[*F* ^2^ > 2σ(*F* ^2^)], *wR*(*F* ^2^), *S*	0.057, 0.177, 1.07
No. of reflections	3039
No. of parameters	178
H-atom treatment	H atoms treated by a mixture of independent and constrained refinement
Δρ_max_, Δρ_min_ (e Å^−3^)	0.17, −0.19

Computer programs: *APEX2* and *SAINT* (Bruker, 2012 ▸), *SHELXS97* (Sheldrick, 2008 ▸), *SHELXL2013* (Sheldrick, 2015 ▸), *Mercury* (Macrae *et al.*, 2006 ▸) and *PLATON* (Spek, 2009 ▸).

## supplementary crystallographic information

### Crystal data

**Table:**

C_24_H_18_O_4_·2C_3_H_7_NO	*Z* = 1
*M**_r_* = 516.57	*F*(000) = 274
Triclinic, *P*1	*D*_x_ = 1.296 Mg m^−^^3^
*a* = 6.0569 (5) Å	Mo *K*α radiation, λ = 0.71073 Å
*b* = 9.5801 (5) Å	Cell parameters from 4190 reflections
*c* = 11.9941 (8) Å	θ = 2.4–23.5°
α = 72.867 (2)°	µ = 0.09 mm^−^^1^
β = 84.649 (2)°	*T* = 294 K
γ = 86.710 (2)°	Block, colourless
*V* = 661.86 (8) Å^3^	0.25 × 0.24 × 0.10 mm

### Data collection

**Table:**

Bruker APEXII DUO CCD area-detector diffractometer	3039 independent reflections
Radiation source: fine-focus sealed tube	1944 reflections with *I* > 2σ(*I*)
Graphite monochromator	*R*_int_ = 0.043
φ and ω scans	θ_max_ = 27.5°, θ_min_ = 1.8°
Absorption correction: multi-scan (SADABS; Bruker, 2012)	*h* = −7→7
*T*_min_ = 0.961, *T*_max_ = 0.991	*k* = −12→12
21963 measured reflections	*l* = −15→15

### Refinement

**Table:**

Refinement on *F*^2^	0 restraints
Least-squares matrix: full	Hydrogen site location: mixed
*R*[*F*^2^ > 2σ(*F*^2^)] = 0.057	H atoms treated by a mixture of independent and constrained refinement
*wR*(*F*^2^) = 0.177	*w* = 1/[σ^2^(*F*_o_^2^) + (0.0694*P*)^2^ + 0.2543*P*] where *P* = (*F*_o_^2^ + 2*F*_c_^2^)/3
*S* = 1.07	(Δ/σ)_max_ < 0.001
3039 reflections	Δρ_max_ = 0.17 e Å^−^^3^
178 parameters	Δρ_min_ = −0.19 e Å^−^^3^

### Special details

**Table:**

Geometry. All esds (except the esd in the dihedral angle between two l.s. planes) are estimated using the full covariance matrix. The cell esds are taken into account individually in the estimation of esds in distances, angles and torsion angles; correlations between esds in cell parameters are only used when they are defined by crystal symmetry. An approximate (isotropic) treatment of cell esds is used for estimating esds involving l.s. planes.

### Fractional atomic coordinates and isotropic or equivalent isotropic displacement parameters (Å^2^)

**Table:**

	*x*	*y*	*z*	*U*_iso_*/*U*_eq_	
O1	−0.1262 (3)	0.5766 (2)	0.23944 (17)	0.0750 (6)	
O2	0.2445 (3)	0.0650 (2)	0.64822 (17)	0.0793 (6)	
H2B	0.108 (7)	0.010 (5)	0.682 (4)	0.145 (15)*	
C1	−0.0487 (4)	0.3283 (3)	0.4223 (2)	0.0545 (6)	
H1A	−0.1859	0.3421	0.3911	0.065*	
C2	−0.0096 (4)	0.2057 (3)	0.5127 (2)	0.0571 (6)	
H2A	−0.1197	0.1376	0.5423	0.069*	
C3	0.1943 (4)	0.1834 (3)	0.5599 (2)	0.0542 (6)	
C4	0.3540 (4)	0.2863 (3)	0.5152 (2)	0.0643 (7)	
H4A	0.4909	0.2727	0.5468	0.077*	
C5	0.3131 (4)	0.4087 (3)	0.4244 (2)	0.0557 (6)	
H5A	0.4233	0.4767	0.3951	0.067*	
C6	0.1110 (3)	0.4324 (2)	0.37597 (18)	0.0460 (5)	
C7	0.0568 (4)	0.5610 (2)	0.2773 (2)	0.0527 (6)	
C8	0.2297 (4)	0.6688 (2)	0.2225 (2)	0.0552 (6)	
H8A	0.3635	0.6600	0.2566	0.066*	
C9	0.2000 (4)	0.7762 (2)	0.1276 (2)	0.0503 (5)	
H9A	0.0634	0.7814	0.0968	0.060*	
C10	0.3566 (3)	0.8892 (2)	0.06404 (18)	0.0452 (5)	
C11	0.5544 (4)	0.9078 (2)	0.1063 (2)	0.0515 (6)	
H11A	0.5925	0.8463	0.1781	0.062*	
C12	0.3042 (4)	0.9840 (2)	−0.0435 (2)	0.0513 (6)	
H12A	0.1716	0.9740	−0.0733	0.062*	
N1	0.3441 (3)	0.2611 (2)	0.16532 (17)	0.0539 (5)	
O3	0.0634 (3)	0.1066 (2)	0.23142 (17)	0.0752 (6)	
C13	0.2500 (4)	0.1452 (3)	0.2375 (2)	0.0610 (6)	
H13A	0.3312	0.0880	0.2977	0.073*	
C14	0.5659 (4)	0.3002 (3)	0.1757 (3)	0.0788 (8)	
H14A	0.6290	0.2276	0.2392	0.118*	
H14B	0.5611	0.3935	0.1906	0.118*	
H14C	0.6553	0.3055	0.1042	0.118*	
C15	0.2266 (5)	0.3526 (3)	0.0692 (3)	0.0767 (8)	
H15A	0.0810	0.3160	0.0728	0.115*	
H15B	0.3065	0.3518	−0.0036	0.115*	
H15C	0.2142	0.4508	0.0746	0.115*	

### Atomic displacement parameters (Å^2^)

**Table:**

	*U*^11^	*U*^22^	*U*^33^	*U*^12^	*U*^13^	*U*^23^
O1	0.0553 (10)	0.0705 (12)	0.0816 (13)	−0.0128 (9)	−0.0211 (9)	0.0120 (10)
O2	0.0605 (11)	0.0750 (13)	0.0769 (13)	−0.0169 (9)	−0.0191 (9)	0.0242 (10)
C1	0.0427 (12)	0.0581 (14)	0.0556 (13)	−0.0102 (10)	−0.0092 (10)	−0.0021 (11)
C2	0.0456 (12)	0.0556 (14)	0.0594 (14)	−0.0185 (10)	−0.0057 (10)	0.0034 (11)
C3	0.0496 (12)	0.0534 (13)	0.0500 (13)	−0.0106 (10)	−0.0049 (10)	0.0018 (10)
C4	0.0446 (12)	0.0718 (17)	0.0646 (15)	−0.0158 (11)	−0.0162 (11)	0.0047 (13)
C5	0.0469 (12)	0.0555 (14)	0.0556 (14)	−0.0185 (10)	−0.0063 (10)	0.0018 (11)
C6	0.0460 (11)	0.0456 (12)	0.0431 (11)	−0.0080 (9)	−0.0033 (9)	−0.0065 (9)
C7	0.0500 (13)	0.0511 (13)	0.0525 (13)	−0.0072 (10)	−0.0079 (10)	−0.0062 (10)
C8	0.0517 (13)	0.0524 (13)	0.0545 (14)	−0.0100 (10)	−0.0106 (10)	−0.0012 (11)
C9	0.0473 (12)	0.0462 (12)	0.0516 (13)	−0.0047 (9)	−0.0045 (9)	−0.0047 (10)
C10	0.0476 (11)	0.0393 (11)	0.0445 (12)	−0.0030 (9)	−0.0027 (9)	−0.0060 (9)
C11	0.0558 (13)	0.0463 (12)	0.0445 (12)	−0.0046 (10)	−0.0115 (10)	0.0018 (9)
C12	0.0496 (12)	0.0490 (13)	0.0511 (13)	−0.0069 (10)	−0.0127 (10)	−0.0047 (10)
N1	0.0451 (10)	0.0505 (11)	0.0621 (12)	−0.0071 (8)	−0.0028 (9)	−0.0097 (9)
O3	0.0622 (11)	0.0740 (12)	0.0797 (13)	−0.0249 (9)	0.0030 (9)	−0.0060 (10)
C13	0.0598 (15)	0.0552 (15)	0.0622 (15)	−0.0043 (12)	−0.0051 (11)	−0.0074 (12)
C14	0.0536 (15)	0.083 (2)	0.104 (2)	−0.0178 (14)	−0.0046 (14)	−0.0301 (17)
C15	0.0751 (18)	0.0691 (18)	0.0739 (19)	−0.0094 (14)	−0.0138 (14)	0.0016 (14)

### Geometric parameters (Å, º)

**Table:**

O1—C7	1.221 (3)	C9—H9A	0.9300
O2—C3	1.347 (3)	C10—C11	1.383 (3)
O2—H2B	0.99 (4)	C10—C12	1.393 (3)
C1—C2	1.370 (3)	C11—C12^i^	1.377 (3)
C1—C6	1.387 (3)	C11—H11A	0.9300
C1—H1A	0.9300	C12—C11^i^	1.377 (3)
C2—C3	1.386 (3)	C12—H12A	0.9300
C2—H2A	0.9300	N1—C13	1.312 (3)
C3—C4	1.377 (3)	N1—C14	1.441 (3)
C4—C5	1.374 (3)	N1—C15	1.445 (3)
C4—H4A	0.9300	O3—C13	1.224 (3)
C5—C6	1.382 (3)	C13—H13A	0.9300
C5—H5A	0.9300	C14—H14A	0.9600
C6—C7	1.481 (3)	C14—H14B	0.9600
C7—C8	1.480 (3)	C14—H14C	0.9600
C8—C9	1.310 (3)	C15—H15A	0.9600
C8—H8A	0.9300	C15—H15B	0.9600
C9—C10	1.466 (3)	C15—H15C	0.9600
			
C3—O2—H2B	110 (2)	C11—C10—C12	117.98 (19)
C2—C1—C6	121.8 (2)	C11—C10—C9	123.14 (19)
C2—C1—H1A	119.1	C12—C10—C9	118.88 (19)
C6—C1—H1A	119.1	C12^i^—C11—C10	121.0 (2)
C1—C2—C3	119.8 (2)	C12^i^—C11—H11A	119.5
C1—C2—H2A	120.1	C10—C11—H11A	119.5
C3—C2—H2A	120.1	C11^i^—C12—C10	121.0 (2)
O2—C3—C4	118.0 (2)	C11^i^—C12—H12A	119.5
O2—C3—C2	123.0 (2)	C10—C12—H12A	119.5
C4—C3—C2	119.0 (2)	C13—N1—C14	122.5 (2)
C5—C4—C3	120.6 (2)	C13—N1—C15	119.9 (2)
C5—C4—H4A	119.7	C14—N1—C15	117.6 (2)
C3—C4—H4A	119.7	O3—C13—N1	124.9 (2)
C4—C5—C6	121.1 (2)	O3—C13—H13A	117.6
C4—C5—H5A	119.4	N1—C13—H13A	117.6
C6—C5—H5A	119.4	N1—C14—H14A	109.5
C5—C6—C1	117.6 (2)	N1—C14—H14B	109.5
C5—C6—C7	123.92 (19)	H14A—C14—H14B	109.5
C1—C6—C7	118.46 (19)	N1—C14—H14C	109.5
O1—C7—C8	120.2 (2)	H14A—C14—H14C	109.5
O1—C7—C6	120.7 (2)	H14B—C14—H14C	109.5
C8—C7—C6	119.10 (19)	N1—C15—H15A	109.5
C9—C8—C7	122.0 (2)	N1—C15—H15B	109.5
C9—C8—H8A	119.0	H15A—C15—H15B	109.5
C7—C8—H8A	119.0	N1—C15—H15C	109.5
C8—C9—C10	127.7 (2)	H15A—C15—H15C	109.5
C8—C9—H9A	116.2	H15B—C15—H15C	109.5
C10—C9—H9A	116.2		
			
C6—C1—C2—C3	−0.1 (4)	C1—C6—C7—C8	−176.6 (2)
C1—C2—C3—O2	−179.5 (2)	O1—C7—C8—C9	−5.6 (4)
C1—C2—C3—C4	0.5 (4)	C6—C7—C8—C9	172.7 (2)
O2—C3—C4—C5	179.4 (2)	C7—C8—C9—C10	−179.5 (2)
C2—C3—C4—C5	−0.6 (4)	C8—C9—C10—C11	−8.4 (4)
C3—C4—C5—C6	0.3 (4)	C8—C9—C10—C12	172.3 (2)
C4—C5—C6—C1	0.0 (4)	C12—C10—C11—C12^i^	−0.5 (4)
C4—C5—C6—C7	−179.3 (2)	C9—C10—C11—C12^i^	−179.8 (2)
C2—C1—C6—C5	−0.1 (4)	C11—C10—C12—C11^i^	0.5 (4)
C2—C1—C6—C7	179.3 (2)	C9—C10—C12—C11^i^	179.8 (2)
C5—C6—C7—O1	−179.0 (2)	C14—N1—C13—O3	−179.1 (3)
C1—C6—C7—O1	1.7 (4)	C15—N1—C13—O3	−0.8 (4)
C5—C6—C7—C8	2.7 (4)		

Symmetry code: (i) −*x*+1, −*y*+2, −*z*.

### Hydrogen-bond geometry (Å, º)

**Table:**

*D*—H···*A*	*D*—H	H···*A*	*D*···*A*	*D*—H···*A*
O2—H2*B*···O3^ii^	0.99 (4)	1.63 (5)	2.592 (3)	162 (4)

Symmetry code: (ii) −*x*, −*y*, −*z*+1.
